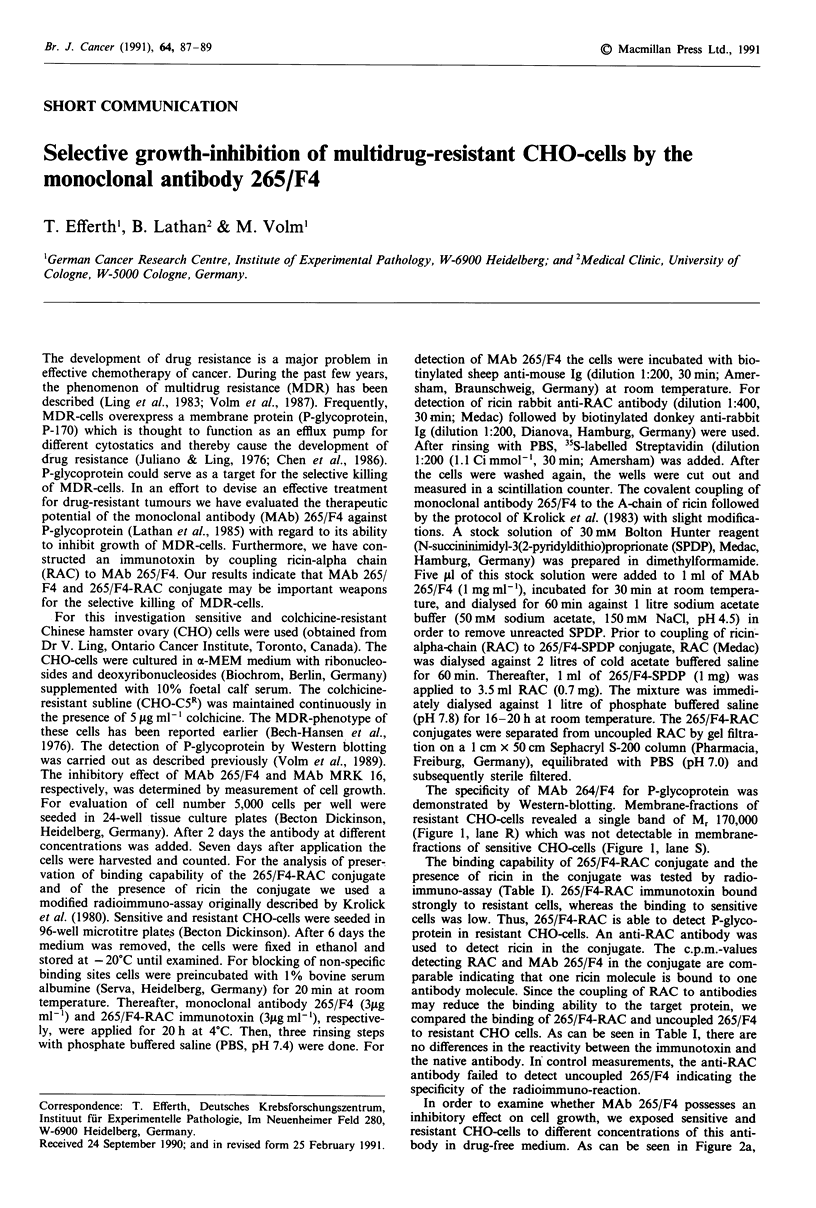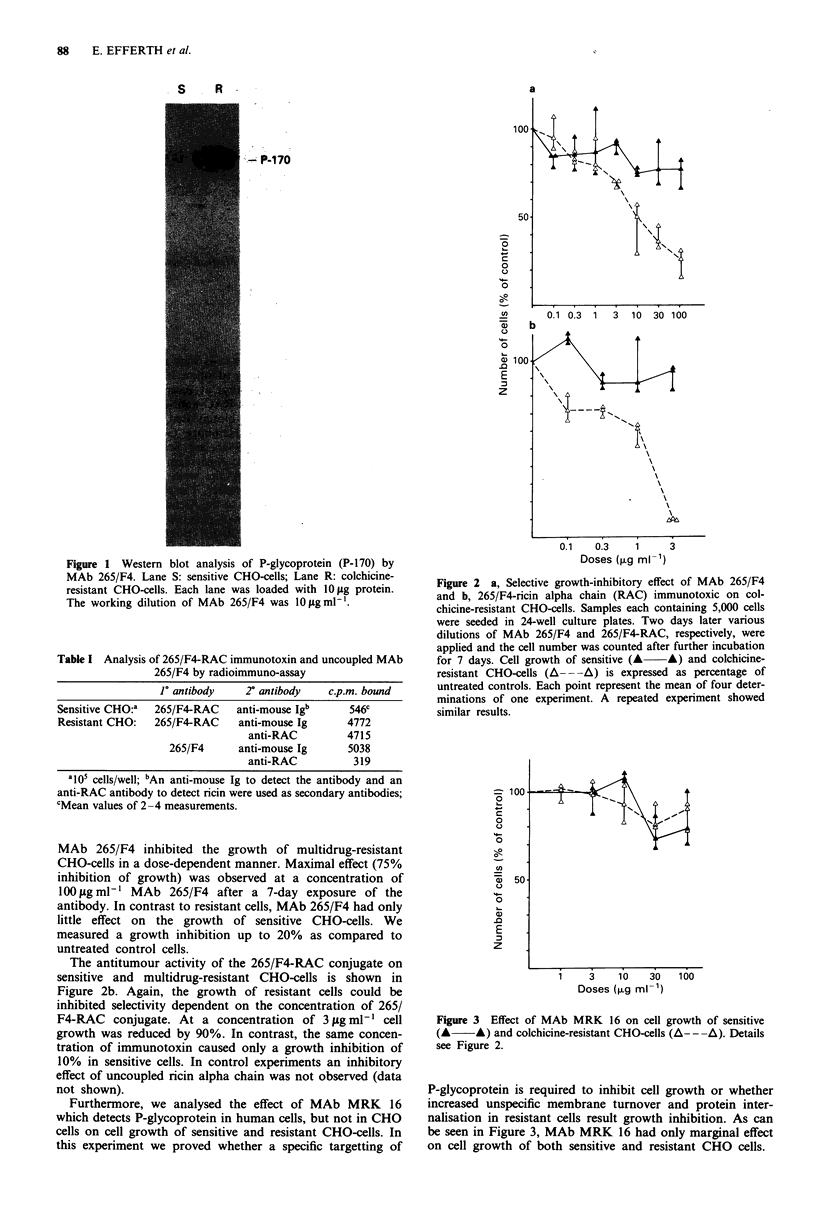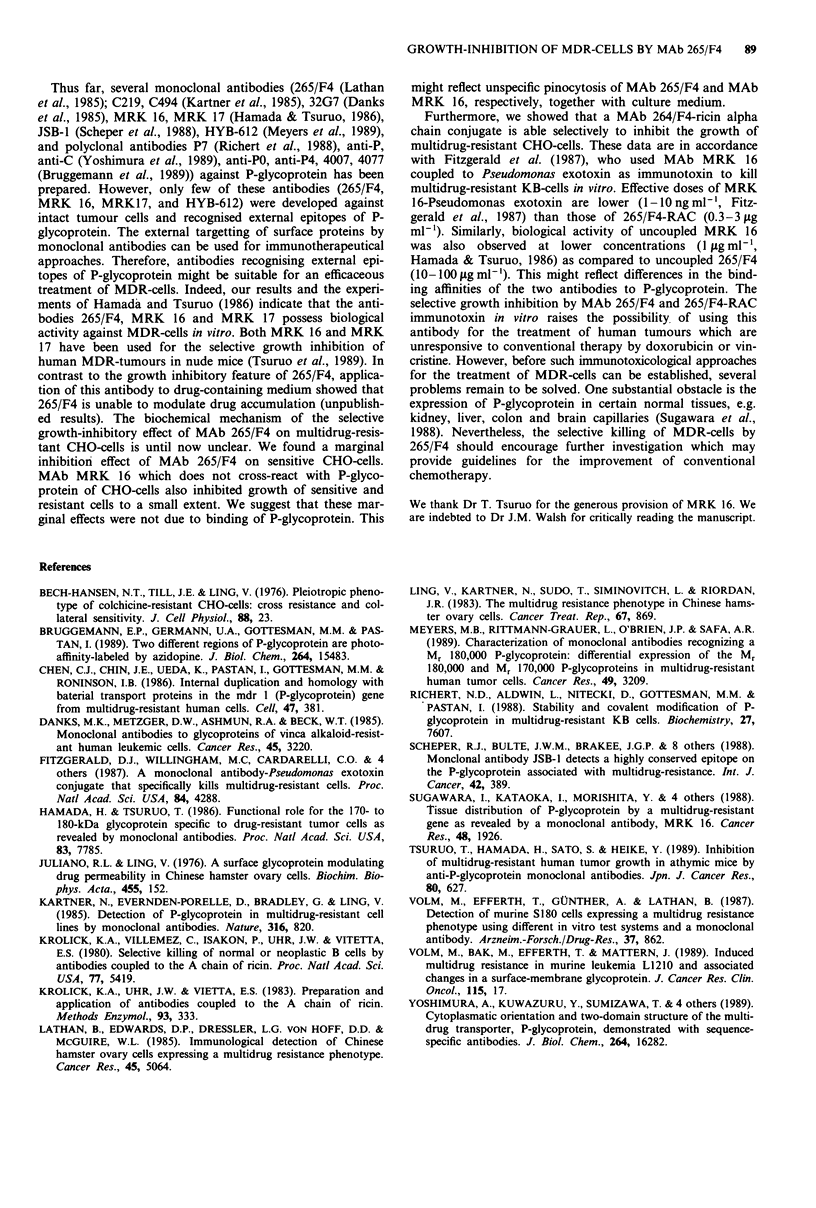# Selective growth-inhibition of multidrug-resistant CHO-cells by the monoclonal antibody 265/F4.

**DOI:** 10.1038/bjc.1991.245

**Published:** 1991-07

**Authors:** T. Efferth, B. Lathan, M. Volm

**Affiliations:** German Cancer Research Centre, Institute of Experimental Pathology, Heidelberg.

## Abstract

**Images:**


Br. J. Cancer (1991), 64, 87-89 © Macmillan Press Ltd., 1991

SHORT COMMUNICATION

### Selective growth-inhibition of multidrug-resistant CHO-cells by the monoclonal antibody 265/F4

T. Efferth[1], B. Lathan[2] & M. Volm[1]

[1]*German Cancer Research Centre, Institute of Experimental Pathology, W-6900 Heidelberg; and* [2]*Medical Clinic, University of Cologne, W-5000 Cologne, Germany.*

The development of drug resistance is a major problem in effective chemotherapy of cancer. During the past few years, the phenomenon of multidrug resistance (MDR) has been described (Ling *et al.*, 1983; Volm *et al.*, 1987). Frequently, MDR-cells overexpress a membrane protein (P-glycoprotein, P-170) which is thought to function as an efflux pump for different cytostatics and thereby cause the development of drug resistance (Juliano & Ling, 1976; Chen *et al.*, 1986). P-glycoprotein could serve as a target for the selective killing of MDR-cells. In an effort to devise an effective treatment for drug-resistant tumours we have evaluated the therapeutic potential of the monoclonal antibody (MAb) 265/F4 against P-glycoprotein (Lathan *et al.*, 1985) with regard to its ability to inhibit growth of MDR-cells. Furthermore, we have constructed an immunotoxin by coupling ricin-alpha chain (RAC) to MAb 265/F4. Our results indicate that MAb 265/F4 and 265/F4-RAC conjugate may be important weapons for the selective killing of MDR-cells.

For this investigation sensitive and colchicine-resistant Chinese hamster ovary (CHO) cells were used (obtained from Dr V. Ling, Ontario Cancer Institute, Toronto, Canada). The CHO-cells were cultured in α-MEM medium with ribonucleosides and deoxyribonucleosides (Biochrom, Berlin, Germany) supplemented with 10% foetal calf serum. The colchicine-resistant subline (CHO-C5$^R$) was maintained continuously in the presence of 5 μg ml$^{-1}$ colchicine. The MDR-phenotype of these cells has been reported earlier (Bech-Hansen *et al.*, 1976). The detection of P-glycoprotein by Western blotting was carried out as described previously (Volm *et al.*, 1989). The inhibitory effect of MAb 265/F4 and MAb MRK 16, respectively, was determined by measurement of cell growth. For evaluation of cell number 5,000 cells per well were seeded in 24-well tissue culture plates (Becton Dickinson, Heidelberg, Germany). After 2 days the antibody at different concentrations was added. Seven days after application the cells were harvested and counted. For the analysis of preservation of binding capability of the 265/F4-RAC conjugate and of the presence of ricin the conjugate we used a modified radioimmuno-assay originally described by Krolick *et al.* (1980). Sensitive and resistant CHO-cells were seeded in 96-well microtitre plates (Becton Dickinson). After 6 days the medium was removed, the cells were fixed in ethanol and stored at −20°C until examined. For blocking of non-specific binding sites cells were preincubated with 1% bovine serum albumine (Serva, Heidelberg, Germany) for 20 min at room temperature. Thereafter, monoclonal antibody 265/F4 (3μg ml$^{-1}$) and 265/F4-RAC immunotoxin (3μg ml$^{-1}$), respectively, were applied for 20 h at 4°C. Then, three rinsing steps with phosphate buffered saline (PBS, pH 7.4) were done. For detection of MAb 265/F4 the cells were incubated with biotinylated sheep anti-mouse Ig (dilution 1:200, 30 min; Amersham, Braunschweig, Germany) at room temperature. For detection of ricin rabbit anti-RAC antibody (dilution 1:400, 30 min; Medac) followed by biotinylated donkey anti-rabbit Ig (dilution 1:200, Dianova, Hamburg, Germany) were used. After rinsing with PBS, $^{35}$S-labelled Streptavidin (dilution 1:200 (1.1 Ci mmol$^{-1}$, 30 min; Amersham) was added. After the cells were washed again, the wells were cut out and measured in a scintillation counter. The covalent coupling of monoclonal antibody 265/F4 to the A-chain of ricin followed by the protocol of Krolick *et al.* (1983) with slight modifications. A stock solution of 30 mM Bolton Hunter reagent (N-succininimidyl-3(2-pyridyldithio)proprionate (SPDP), Medac, Hamburg, Germany) was prepared in dimethylformamide. Five μl of this stock solution were added to 1 ml of MAb 265/F4 (1 mg ml$^{-1}$), incubated for 30 min at room temperature, and dialysed for 60 min against 1 litre sodium acetate buffer (50 mM sodium acetate, 150 mM NaCl, pH 4.5) in order to remove unreacted SPDP. Prior to coupling of ricin-alpha-chain (RAC) to 265/F4-SPDP conjugate, RAC (Medac) was dialysed against 2 litres of cold acetate buffered saline for 60 min. Thereafter, 1 ml of 265/F4-SPDP (1 mg) was applied to 3.5 ml RAC (0.7 mg). The mixture was immediately dialysed against 1 litre of phosphate buffered saline (pH 7.8) for 16–20 h at room temperature. The 265/F4-RAC conjugates were separated from uncoupled RAC by gel filtration on a 1 cm × 50 cm Sephacryl S-200 column (Pharmacia, Freiburg, Germany), equilibrated with PBS (pH 7.0) and subsequently sterile filtered.

The specificity of MAb 264/F4 for P-glycoprotein was demonstrated by Western-blotting. Membrane-fractions of resistant CHO-cells revealed a single band of $M_r$ 170,000 (Figure 1, lane R) which was not detectable in membrane-fractions of sensitive CHO-cells (Figure 1, lane S).

The binding capability of 265/F4-RAC conjugate and the presence of ricin in the conjugate was tested by radioimmuno-assay (Table I). 265/F4-RAC immunotoxin bound strongly to resistant cells, whereas the binding to sensitive cells was low. Thus, 265/F4-RAC is able to detect P-glycoprotein in resistant CHO-cells. An anti-RAC antibody was used to detect ricin in the conjugate. The c.p.m.-values detecting RAC and MAb 265/F4 in the conjugate are comparable indicating that one ricin molecule is bound to one antibody molecule. Since the coupling of RAC to antibodies may reduce the binding ability to the target protein, we compared the binding of 265/F4-RAC and uncoupled 265/F4 to resistant CHO cells. As can be seen in Table I, there are no differences in the reactivity between the immunotoxin and the native antibody. In control measurements, the anti-RAC antibody failed to detect uncoupled 265/F4 indicating the specificity of the radioimmuno-reaction.

In order to examine whether MAb 265/F4 possesses an inhibitory effect on cell growth, we exposed sensitive and resistant CHO-cells to different concentrations of this antibody in drug-free medium. As can be seen in Figure 2a,

Correspondence: T. Efferth, Deutsches Krebsforschungszentrum, Instituut für Experimentelle Pathologie, Im Neuenheimer Feld 280, W-6900 Heidelberg, Germany.
Received 24 September 1990; and in revised form 25 February 1991.

**Figure 1** Western blot analysis of P-glycoprotein (P-170) by MAb 265/F4. Lane S: sensitive CHO-cells; Lane R: colchicine-resistant CHO-cells. Each lane was loaded with 10 μg protein. The working dilution of MAb 265/F4 was $10\,\mu g\,ml^{-1}$.

**Figure 2** **a**, Selective growth-inhibitory effect of MAb 265/F4 and **b**, 265/F4-ricin alpha chain (RAC) immunotoxic on colchicine-resistant CHO-cells. Samples each containing 5,000 cells were seeded in 24-well culture plates. Two days later various dilutions of MAb 265/F4 and 265/F4-RAC, respectively, were applied and the cell number was counted after further incubation for 7 days. Cell growth of sensitive (▲——▲) and colchicine-resistant CHO-cells (Δ---Δ) is expressed as percentage of untreated controls. Each point represent the mean of four determinations of one experiment. A repeated experiment showed similar results.

**Table I** Analysis of 265/F4-RAC immunotoxin and uncoupled MAb 265/F4 by radioimmuno-assay

| | *1° antibody* | *2° antibody* | *c.p.m. bound* |
|---|---|---|---|
| Sensitive CHO:[a] | 265/F4-RAC | anti-mouse Ig[b] | 546[c] |
| Resistant CHO: | 265/F4-RAC | anti-mouse Ig | 4772 |
| | | anti-RAC | 4715 |
| | 265/F4 | anti-mouse Ig | 5038 |
| | | anti-RAC | 319 |

[a]$10^5$ cells/well; [b]An anti-mouse Ig to detect the antibody and an anti-RAC antibody to detect ricin were used as secondary antibodies; [c]Mean values of 2–4 measurements.

**Figure 3** Effect of MAb MRK 16 on cell growth of sensitive (▲——▲) and colchicine-resistant CHO-cells (Δ---Δ). Details see Figure 2.

MAb 265/F4 inhibited the growth of multidrug-resistant CHO-cells in a dose-dependent manner. Maximal effect (75% inhibition of growth) was observed at a concentration of $100\,\mu g\,ml^{-1}$ MAb 265/F4 after a 7-day exposure of the antibody. In contrast to resistant cells, MAb 265/F4 had only little effect on the growth of sensitive CHO-cells. We measured a growth inhibition up to 20% as compared to untreated control cells.

The antitumour activity of the 265/F4-RAC conjugate on sensitive and multidrug-resistant CHO-cells is shown in Figure 2b. Again, the growth of resistant cells could be inhibited selectivity dependent on the concentration of 265/F4-RAC conjugate. At a concentration of $3\,\mu g\,ml^{-1}$ cell growth was reduced by 90%. In contrast, the same concentration of immunotoxin caused only a growth inhibition of 10% in sensitive cells. In control experiments an inhibitory effect of uncoupled ricin alpha chain was not observed (data not shown).

Furthermore, we analysed the effect of MAb MRK 16 which detects P-glycoprotein in human cells, but not in CHO cells on cell growth of sensitive and resistant CHO-cells. In this experiment we proved whether a specific targetting of P-glycoprotein is required to inhibit cell growth or whether increased unspecific membrane turnover and protein internalisation in resistant cells result growth inhibition. As can be seen in Figure 3, MAb MRK 16 had only marginal effect on cell growth of both sensitive and resistant CHO cells.

Thus far, several monoclonal antibodies (265/F4 (Lathan *et al.*, 1985); C219, C494 (Kartner *et al.*, 1985), 32G7 (Danks *et al.*, 1985), MRK 16, MRK 17 (Hamada & Tsuruo, 1986), JSB-1 (Scheper *et al.*, 1988), HYB-612 (Meyers *et al.*, 1989), and polyclonal antibodies P7 (Richert *et al.*, 1988), anti-P, anti-C (Yoshimura *et al.*, 1989), anti-P0, anti-P4, 4007, 4077 (Bruggemann *et al.*, 1989)) against P-glycoprotein has been prepared. However, only few of these antibodies (265/F4, MRK 16, MRK17, and HYB-612) were developed against intact tumour cells and recognised external epitopes of P-glycoprotein. The external targetting of surface proteins by monoclonal antibodies can be used for immunotherapeutical approaches. Therefore, antibodies recognising external epitopes of P-glycoprotein might be suitable for an efficaceous treatment of MDR-cells. Indeed, our results and the experiments of Hamada and Tsuruo (1986) indicate that the antibodies 265/F4, MRK 16 and MRK 17 possess biological activity against MDR-cells *in vitro*. Both MRK 16 and MRK 17 have been used for the selective growth inhibition of human MDR-tumours in nude mice (Tsuruo *et al.*, 1989). In contrast to the growth inhibitory feature of 265/F4, application of this antibody to drug-containing medium showed that 265/F4 is unable to modulate drug accumulation (unpublished results). The biochemical mechanism of the selective growth-inhibitory effect of MAb 265/F4 on multidrug-resistant CHO-cells is until now unclear. We found a marginal inhibition effect of MAb 265/F4 on sensitive CHO-cells. MAb MRK 16 which does not cross-react with P-glycoprotein of CHO-cells also inhibited growth of sensitive and resistant cells to a small extent. We suggest that these marginal effects were not due to binding of P-glycoprotein. This might reflect unspecific pinocytosis of MAb 265/F4 and MAb MRK 16, respectively, together with culture medium.

Furthermore, we showed that a MAb 264/F4-ricin alpha chain conjugate is able selectively to inhibit the growth of multidrug-resistant CHO-cells. These data are in accordance with Fitzgerald *et al.* (1987), who used MAb MRK 16 coupled to *Pseudomonas* exotoxin as immunotoxin to kill multidrug-resistant KB-cells *in vitro*. Effective doses of MRK 16-Pseudomonas exotoxin are lower ($1-10\,ng\,ml^{-1}$, Fitzgerald *et al.*, 1987) than those of 265/F4-RAC ($0.3-3\,\mu g\,ml^{-1}$). Similarly, biological activity of uncoupled MRK 16 was also observed at lower concentrations ($1\,\mu g\,ml^{-1}$, Hamada & Tsuruo, 1986) as compared to uncoupled 265/F4 ($10-100\,\mu g\,ml^{-1}$). This might reflect differences in the binding affinities of the two antibodies to P-glycoprotein. The selective growth inhibition by MAb 265/F4 and 265/F4-RAC immunotoxin *in vitro* raises the possibility of using this antibody for the treatment of human tumours which are unresponsive to conventional therapy by doxorubicin or vincristine. However, before such immunotoxicological approaches for the treatment of MDR-cells can be established, several problems remain to be solved. One substantial obstacle is the expression of P-glycoprotein in certain normal tissues, e.g. kidney, liver, colon and brain capillaries (Sugawara *et al.*, 1988). Nevertheless, the selective killing of MDR-cells by 265/F4 should encourage further investigation which may provide guidelines for the improvement of conventional chemotherapy.

We thank Dr T. Tsuruo for the generous provision of MRK 16. We are indebted to Dr J.M. Walsh for critically reading the manuscript.

### References

BECH-HANSEN, N.T., TILL, J.E. & LING, V. (1976). Pleiotropic phenotype of colchicine-resistant CHO-cells: cross resistance and collateral sensitivity. *J. Cell Physiol.*, **88**, 23.

BRUGGEMANN, E.P., GERMANN, U.A., GOTTESMAN, M.M. & PASTAN, I. (1989). Two different regions of P-glycoprotein are photo-affinity-labeled by azidopine. *J. Biol. Chem.*, **264**, 15483.

CHEN, C.J., CHIN, J.E., UEDA, K., PASTAN, I., GOTTESMAN, M.M. & RONINSON, I.B. (1986). Internal duplication and homology with baterial transport proteins in the mdr 1 (P-glycoprotein) gene from multidrug-resistant human cells. *Cell*, **47**, 381.

DANKS, M.K., METZGER, D.W., ASHMUN, R.A. & BECK, W.T. (1985). Monoclonal antibodies to glycoproteins of vinca alkaloid-resistant human leukemic cells. *Cancer Res.*, **45**, 3220.

FITZGERALD, D.J., WILLINGHAM, M.C, CARDARELLI, C.O. & 4 others (1987). A monoclonal antibody-*Pseudomonas* exotoxin conjugate that specifically kills multidrug-resistant cells. *Proc. Natl Acad. Sci. USA*, **84**, 4288.

HAMADA, H. & TSURUO, T. (1986). Functional role for the 170- to 180-kDa glycoprotein specific to drug-resistant tumor cells as revealed by monoclonal antibodies. *Proc. Natl Acad. Sci. USA*, **83**, 7785.

JULIANO, R.L. & LING, V. (1976). A surface glycoprotein modulating drug permeability in Chinese hamster ovary cells. *Biochim. Biophys. Acta.*, **455**, 152.

KARTNER, N., EVERNDEN-PORELLE, D., BRADLEY, G. & LING, V. (1985). Detection of P-glycoprotein in multidrug-resistant cell lines by monoclonal antibodies. *Nature*, **316**, 820.

KROLICK, K.A., VILLEMEZ, C., ISAKON, P., UHR, J.W. & VITETTA, E.S. (1980). Selective killing of normal or neoplastic B cells by antibodies coupled to the A chain of ricin. *Proc. Natl Acad. Sci. USA*, **77**, 5419.

KROLICK, K.A., UHR, J.W. & VIETTA, E.S. (1983). Preparation and application of antibodies coupled to the A chain of ricin. *Methods Enzymol.*, **93**, 333.

LATHAN, B., EDWARDS, D.P., DRESSLER, L.G. VON HOFF, D.D. & MCGUIRE, W.L. (1985). Immunological detection of Chinese hamster ovary cells expressing a multidrug resistance phenotype. *Cancer Res.*, **45**, 5064.

LING, V., KARTNER, N., SUDO, T., SIMINOVITCH, L. & RIORDAN, J.R. (1983). The multidrug resistance phenotype in Chinese hamster ovary cells. *Cancer Treat. Rep.*, **67**, 869.

MEYERS, M.B., RITTMANN-GRAUER, L., O'BRIEN, J.P. & SAFA, A.R. (1989). Characterization of monoclonal antibodies recognizing a $M_r$ 180,000 P-glycoprotein: differential expression of the $M_r$ 180,000 and $M_r$ 170,000 P-glycoproteins in multidrug-resistant human tumor cells. *Cancer Res.*, **49**, 3209.

RICHERT, N.D., ALDWIN, L., NITECKI, D., GOTTESMAN, M.M. & PASTAN, I. (1988). Stability and covalent modification of P-glycoprotein in multidrug-resistant KB cells. *Biochemistry*, **27**, 7607.

SCHEPER, R.J., BULTE, J.W.M., BRAKEE, J.G.P. & 8 others (1988). Monclonal antibody JSB-1 detects a highly conserved epitope on the P-glycoprotein associated with multidrug-resistance. *Int. J. Cancer*, **42**, 389.

SUGAWARA, I., KATAOKA, I., MORISHITA, Y. & 4 others (1988). Tissue distribution of P-glycoprotein by a multidrug-resistant gene as revealed by a monoclonal antibody, MRK 16. *Cancer Res.*, **48**, 1926.

TSURUO, T., HAMADA, H., SATO, S. & HEIKE, Y. (1989). Inhibition of multidrug-resistant human tumor growth in athymic mice by anti-P-glycoprotein monoclonal antibodies. *Jpn. J. Cancer Res.*, **80**, 627.

VOLM, M., EFFERTH, T., GÜNTHER, A. & LATHAN, B. (1987). Detection of murine S180 cells expressing a multidrug resistance phenotype using different in vitro test systems and a monoclonal antibody. *Arzneim.-Forsch./Drug-Res.*, **37**, 862.

VOLM, M., BAK, M., EFFERTH, T. & MATTERN, J. (1989). Induced multidrug resistance in murine leukemia L1210 and associated changes in a surface-membrane glycoprotein. *J. Cancer Res. Clin. Oncol.*, **115**, 17.

YOSHIMURA, A., KUWAZURU, Y., SUMIZAWA, T. & 4 others (1989). Cytoplasmatic orientation and two-domain structure of the multidrug transporter, P-glycoprotein, demonstrated with sequence-specific antibodies. *J. Biol. Chem.*, **264**, 16282.